# Transcriptomics of two evolutionary novelties: how to make a sperm-transfer organ out of an anal fin and a sexually selected “sword” out of a caudal fin

**DOI:** 10.1002/ece3.1390

**Published:** 2015-01-23

**Authors:** Ji Hyoun Kang, Tereza Manousaki, Paolo Franchini, Susanne Kneitz, Manfred Schartl, Axel Meyer

**Affiliations:** 1 Lehrstuhl für Zoologie und Evolutionsbiologie, Department of Biology, University of KonstanzUniversitätsstraβe 10, 78457, Konstanz, Germany; 2 Konstanz Research School Chemical Biology, University of KonstanzKonstanz, Germany; 3 Institute of Marine Biology, Biotechnology and Aquaculture, Hellenic Centre for Marine ResearchHeraklion, Greece; 4 Physiological Chemistry, Biozentrum, University of WürzburgAm Hubland, Würzburg, Germany; 5 Comprehensive Cancer Center, University Clinic WürzburgJosef Schneider Straβe 6, 97074, Würzburg, Germany

**Keywords:** Co-option, gonopodium, key innovation, male-specific traits, RNA-Seq, swordtails, *Xiphophorus*

## Abstract

Swords are exaggerated male ornaments of swordtail fishes that have been of great interest to evolutionary biologists ever since Darwin described them in the Descent of Man (1871). They are a novel sexually selected trait derived from modified ventral caudal fin rays and are only found in the genus *Xiphophorus*. Another phylogenetically more widespread and older male trait is the gonopodium, an intromittent organ found in all poeciliid fishes, that is derived from a modified anal fin. Despite many evolutionary and behavioral studies on both traits, little is known so far about the molecular mechanisms underlying their development. By investigating transcriptomic changes (utilizing a RNA-Seq approach) in response to testosterone treatment in the swordtail fish, *Xiphophorus hellerii*, we aimed to better understand the architecture of the gene regulatory networks underpinning the development of these two evolutionary novelties. Large numbers of genes with tissue-specific expression patterns were identified. Among the “sword genes” those involved in embryonic organ development, sexual character development and coloration were highly expressed, while in the gonopodium rather more morphogenesis-related genes were found. Interestingly, many genes and genetic pathways are shared between both developing novel traits derived from median fins: the sword and the gonopodium. Our analyses show that a larger set of gene networks was co-opted during the development and evolution of the “older” gonopodium than in the “younger,” and morphologically less complex trait, the sword. We provide a catalog of candidate genes for future efforts to dissect the development of those sexually selected exaggerated male traits in swordtails.

## Introduction

Exaggerated male-specific traits, or exaggerated secondary sexual characters, are known from many animals and are of great interest to evolutionary biologists. For example, the peacock's tail, the eye-span of stalk-eyed flies, and the feather ornaments of flycatchers are all believed to be products of intersexual selection, as they are attractive to females (reviewed in Andersson 1994). Females are known to base their mate choice on those traits, and this will tend to drive their evolution toward becoming more and more exaggerated (reviewed in Andersson 1994).

The “sword” in the genus *Xiphophorus* (Family: Poeciliidae) is a famous example that was already known to Darwin (1871) of a sexually selected male-specific trait. This trait is favored by females although the preference is not fixed in all species (Wong and Rosenthal 2006). The sword is composed of extended colorful ventral caudal fin rays, and it characterizes several species of the genus *Xiphophorus*. Species of this genus that carry swords are called swordtails, while nonsworded species are colloquially referred to as platyfish. Within swordtails, the swords vary: some species have very long extended colorful swords that can be longer than the body of the males, whereas others have only short and colorless ventral protrusions in the caudal fin (Rosen 1960; Kallman and Kazianis 2006). Some, swordtails that also tend to have more slender bodies than platies, for example, *X. pygmaeus*, and *X. continens*, do not have much of a sword at all (Meyer et al. 1994; Meyer 1997). In several *Xiphophorus* species, females prefer males with longer swords, and even females in platyfish, whose males do not have swords, are attracted to conspecific males with artificial longer swords and sworded males of other species over their own nonsworded conspecific males (Gordon and Rosen 1951; Basolo 1990a, 1991, 1995a,b). The sword has evoked important questions relating to its origin and subsequent evolution such as the pre-existing female bias hypothesis (Basolo 1990b, 1995a) and the role of this sexually selected trait in hybridization-driven speciation in this fish group (Meyer et al. 1994, 2006; Jones et al. 2012, 2013; Kang et al. 2013). Although a hybrid origin of species might be expected to be rare, in fish other instances are known, for example also from cichlid fish (Rüber et al. 2001). Moreover, one might expect that speciation is accompanied by transcriptomic changes in addition to changes in copy numbers of genes and positive selection acting (Steinke et al. 2006; Elmer et al. 2010). Recent comprehensive phylogenetic analyses, using different types of molecular markers, have consistently revealed that this evolutionary novelty has arisen once during the diversification of the genus *Xiphophorus* from ancestral poeciliids, but, interestingly, has been independently lost multiple times (Meyer et al. 1994, 2006; Meyer 1997; Kang et al. 2013).

Another male-specific trait found in all species in the genus *Xiphophorus* is the gonopodium. It is derived from another modified median fin, the anal fin and is made up by its heavily modified rays 3, 4, and 5. They are transformed into this sperm-transferring intromittent organ that is used in copulation in the viviparous poeciliid fish family (Langer 1913; Parenti 1981). While the sword is an evolutionary innovation that is exclusively restricted to the genus *Xiphophorus* (a colorless “sword” is also found in *Poecilia petenensis*), the gonopodium is an evolutionarily older and phylogenetically more widespread trait that is found in all poeciliid fishes. The gonopodium develops species-specific terminal structures such as hooks, spines, and a claw that are believed to serve as a natural mating barrier (in a lock-and-key type) between species, although the proposed prezygotic isolating mechanism has not been shown to act particularly stringently, and hybridization is known to occur among some closely related species (Gordon and Rosen 1951; Rosen and Bailey 1963).

Despite numerous phylogenetic and behavioral studies that aimed to study the evolutionary history and behavioral roles in determining mating success of the sword and gonopodium, much less is known about the genetic basis underlying the development of these traits. Interestingly, the induction of the development of the gonopodium by exogenous testosterone has been demonstrated in juveniles, adult females (Grobstein 1947, 1948; Zauner et al. 2003; Offen et al. 2009, 2013), and even another poeciliid fish, *Gambusia affinis* (Turner 1947; Angus et al. 2001; Ogino et al. 2004). Testosterone increases outgrowth of the gonopodium in a concentration-dependent manner in *X. hellerii* (Offen et al. 2013). Exogenous testosterone can also induce swords or sword-like protrusions from ventral caudal fins in several swordtail and platyfish species (Dzwillo 1964; Zauner et al. 2003; Yanong et al. 2006). All these pieces of evidence imply that androgen signaling is involved in the hormone-induced sword and gonopodial development (Offen et al. 2013). However, the signaling pathways have not been investigated at the molecular level, and our previous work aimed to find other molecular mechanisms potentially involved in the development of those two male-specific traits. Candidate gene approaches revealed that *msxC* and *fgfr1* are upregulated in developing sword and gonopodium under testosterone treatment (Zauner et al. 2003; Offen et al. 2008). In a previous study using suppressive subtractive hybridization (SHH), genes or pathways related to sword and gonopodial development were found, and we discovered that over 100 genes are involved during the development of both traits in *X. hellerii* (128 genes were up- and downregulated in the development of sword and gonopodium) (Offen et al. 2009). However, these findings were based on a cDNA library of *X. hellerii,* and differentially expressed genes were detected in the pooled gonopodium and sword tissues. Gonopodium- or sword-specific genes could hence not be discriminated. Exploring gene expression in the development of the gonopodium and the sword separately to determine their specific roles in terms of developmental molecular mechanisms was the aim of this study.

### Evolutionary novelties: their evolutionary origins and their developmental bases

Identification of the genes or pathways regulating the transformation of the anal and caudal fin into a gonopodium and the development of a sword during sexual maturation provide a key step to understanding the molecular mechanisms leading to evolutionary innovations such as gains or losses of the sword or intraspecific variation in gonopodium morphology. How evolutionary novel traits arise remains an open question in evolutionary biology. There are several definitions for “novel traits” or “evolutionary novelties,” yet they are generally seen as structures that are neither homologous to any structure in ancestral species nor homonomous to any other structures of the same organism (Müller and Wagner 1991). Evolutionary novelties can be categorized based on their novel functional capabilities (e.g., flight or vision) or structural elements (e.g., hair and horn in mammals, scales in reptiles) by focusing on the developmental origin of novel body parts (Wagner and Lynch 2010). The investigation of the origin and divergence of the novel gene regulatory networks contributing to morphological innovations would thereby also allow to better understand both their unique developmental and their evolutionary identities (Wagner and Lynch 2010).

Here, we performed a transcriptome-wide expression analysis to study the molecular pathways involved in the hormone-induced sword and gonopodium development in *X. hellerii*. Through RNA-Seq, we identified a large number of differentially expressed genes during the development of the sword and the metamorphosis of the anal fin into a gonopodium. These analyses help to increase our understanding of the molecular processes underlying the ontogeny and phylogeny of both of the sword, and the gonopodium and provide the foundation for future studies on the molecular mechanisms that led to the evolutionary origin of these evolutionary key innovations.

## Materials and Methods

### Fish and hormone treatments

To study the molecular pathways involved in the development of sword and gonopodium in *X. hellerii,* we testosterone-treated immature juveniles to reach the same ontogenetic stages of the development of sword and gonopodium. We chose this experimental design because the timing of sword and gonopodium during sexual maturation and consequent development of secondary sex characters vary among individuals. This approach allowed for better capturing of transcriptional changes during the development of both of those traits by hormonally inducing identical development in all individuals. It is known that no sex-related morphological differences are found in hormonally induced swords in immature juveniles (Dzwillo 1964; Zander and Dzwillo 1969).

Pregnant females of *X. hellerii* (Konstanz laboratory strain) were taken from stocks kept at the animal research facility at the University of Konstanz. The fish were maintained on a 12 h:12 h light:dark cycle at 24°C in 110-L, densely planted aquaria and were fed with TetraMin flake food and *Artemia* nauplii. A single pregnant female was chosen and kept separately in a 40-L tank until she gave birth, and her juveniles (i.e., one brood) were raised up to 3 months of age under the same conditions. Juvenile individuals (*n* = 8; total length: 2.5–2.7 cm) from the same brood were divided into two groups: testosterone treatment (*n* = 4) and mock treatment (*n* = 4). 17-*α*-methyltestosterone dissolved in ethanol was added to the water of the treatment group. Either testosterone or mock treatment (ethanol) was repeated at day 4 after the initial treatment to maintain the effect of testosterone. We chose a final concentration of 10 *μ*g/L of testosterone as at this concentration the induction of sword/gonopodium development was successful in our other previous studies (Offen et al. 2008, 2009, 2013). A short period of time for treatment (e.g., 1–2 days or even shorter) might be enough for the detection of gene expression differences that are involved in the induction of sword/gonopodium development (more direct target). Although the timing of onset of expression of genes related to sword and gonopodium development after testosterone treatment has not yet been explored in detail, the changes in the expression level of many genes in the fin rays in the hormone-treated fish were detected at 5 days of treatment previously (Offen et al. 2009). Furthermore, morphological changes (i.e., outgrowth of fin rays) of anal (gonopodium) and ventral caudal (sword) fins in the hormone-treated fish became clearly visible at 5 days.

**Figure 1:**
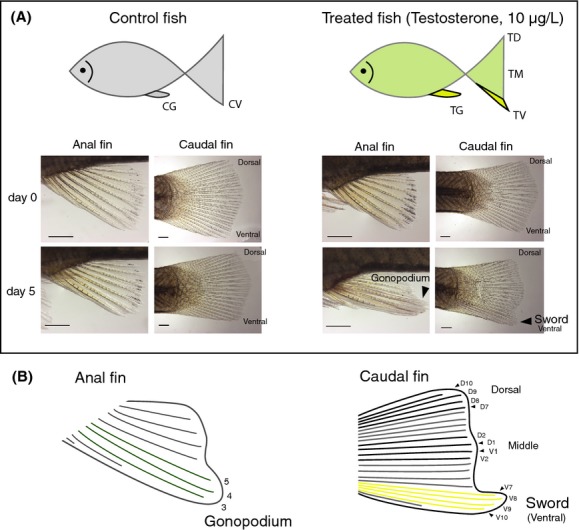
Anal and caudal fins in the control and treated fish at day 0 and day 5 (A) and details of fin rays in the developing sword and gonopodium (B). (A) In the control fish, no difference between anal fin (CG) and ventral caudal fin (CV) was observed at day 0 and day 5 (scale bar = 1 mm). In testosterone-treated fish, initiation of the transformation into a gonopodium (TG) and sword (TV) was apparent at day 5. (B) The gonopodium developed from rays 3, 4, and 5 in anal fin, and the sword developed from V7 to V10 (ventral). Tissues from dorsal rays (D7–D10) and middle rays (V1, V2, D1 and D2) were used for RNA sequencing. *T stands for treated, C for nontreated (control), V for ventral caudal rays (sword), M for middle caudal rays, D for dorsal caudal rays.

At day 5, fish were anaesthetized by incubation in a solution of 0.08 mg/mL tricaine (3-aminobenzoicacid-ethylester-methanesulfonate; Sigma, Munich, Germany). Anal (rays 3–5) and dorsal (D7–V10), middle (D1, D2, V1, and V2) and ventral (V7–V10) rays of the caudal fin from testosterone-treated fish were amputated using a sterile razor blade. Only the anal fin (rays 3–5) and ventral (V7–V10) rays of caudal fin were used for mock-treated control fish. The structures of fin rays used in the transcriptomic experiments are shown in Fig.1.

### RNA extraction

Total RNA of each fin tissue was isolated with Trizol (Invitrogen, Darmstadt, Germany). Tissues were homogenized using pestles and chloroform extraction. RNA was further purified using RNeasy columns (Qiagen, Stockach, Germany). Then on-column DNase treatment was performed according to the manufacturer's protocols (Qiagen, Stockach, Germany). In additional washing and drying steps, we washed columns twice with 80% EtOH to remove all traces of salt and ethanol, and spun them dry for 5 min. RNA was eluted in RNase- and DNase-free water. RNA purity was assessed by a Nanodrop (Thermo Scientific, Wilmington, Germany), and RNA integrity was assessed using a Bioanalyzer 2100 (Agilent, Waldbronn, Germany).

### Library construction and sequencing

Total RNAs recovered from the tissue of dorsal/middle/ventral caudal and anal fin rays in testosterone-treated and nontreated control fish were subjected to high-throughput transcriptome sequencing (RNA-Seq). For testosterone-treated fish, 16 cDNA libraries (four tissues × four individuals) were constructed: treated dorsal caudal fin (TD), treated middle caudal fin (TM), treated ventral caudal fin (“developing sword”: TV), and treated anal fin (“developing gonopodium”: TG) (see Fig.1). For control fish, 8 cDNA libraries (two tissues × four individuals) were constructed only from two tissues including untreated ventral caudal fin (CV) and untreated anal fin (CG) (see Fig.1). Sequencing libraries were constructed using the Illumina TruSeq RNA sample preparation kit (low-throughput protocol) according to the manufacturer's instructions (Illumina, San Diego, USA). Briefly, 500 ng of RNA was subjected to mRNA selection using poly-T oligo-attached magnetic beads followed by chemical fragmentation (5 min, 94°C). The cleaved RNA fragments were then copied into first-strand cDNA using SuperScript II reverse transcriptase (Invitrogen, Darmstadt, Germany) and Illumina proprietary random hexamer primers. After second-strand synthesis using Illumina-supplied consumables, the cDNA was amplified with reagents of the same kit according to the manufacturer's protocols and ligated to bar-coded adapters. The final libraries were amplified using 15 PCR cycles. Quality assessment of the libraries was performed on a Bioanalyzer 2100 (Agilent, Waldbronn, Germany), and the quantification was carried out in the Qubit 2.0 fluorometer (Life Technologies, Darmstadt, Germany). The 24 bar-coded samples were equimolar-pooled and the same pool was loaded on three different lanes of an Illumina flowcell in order to obtain technical replications as well as considerable sequencing depth. Paired-end sequencing of clustered template DNA was performed in the University of Konstanz Genomics Center (GeCKo) on a Genome Analyzer IIx using four-color DNA sequencing-by-synthesis (SBS) technology with 151 cycles (72 cycles for each paired-read and seven cycles for the barcode sequences).

### Raw reads and quality filtering

After sequencing, we obtained 272,288,416 raw reads that were quality-controlled before assembly, read mapping, and downstream analyses. First, the remaining adapters were removed with SeqPrep (https://github.com/jstjohn/SeqPrep), and the overlapping paired-reads were merged. Quality of the sequences was assessed with FastQC (http://www.bioinformatics.bbsrc.ac.uk/projects/fastqc/), and the reads were trimmed further in CLC Genomics Workbench v4.9 (CLC bio, Aarhus, Denmark). Low-quality reads (CLC parameter “limit” set to 0.05) and reads shorter than 20 nucleotides were excluded. Finally, we obtained a total of 201,148,252 filtered reads (5,655,070–11,861,043 reads per sample) with a mean length of 53 nucleotides.

### Transcriptome assembly

Two strategies were employed for producing the assembly: a de novo assembly with Velvet 1.2.07/Oases 0.2.08 (Zerbino and Birney 2008; Schulz et al. 2012) and a “reference-based” assembly using the genome of the closely related species *X. maculatus* as a reference (*Xiphophorus maculatus*-4.4.2, GenBank Assembly ID: GCA_000241075.1) (Schartl et al. 2013). For the de novo assembly, we used a series of kmer values (21–59 with a step of 2) and merged the produced assemblies as described in the Oases manual. We obtained 23,047 loci (including 247,959 transcripts). The reference-based assembly was produced by aligning the reads to the *X. maculatus* genome using Bowtie2 (Langmead and Salzberg 2012), and the transcripts were produced using Cufflinks 2.0.2 (Trapnell et al. 2012). The resulting reference-based assembly contained 47,812 transcripts assigned to 41,360 different loci. To evaluate the two independent assemblies, a BLASTX (Altschul et al. 1997) search was conducted against the stickleback protein data set of Ensembl v68 (Flicek et al. 2012) with e-value cut-off of 10^−6^. The comparison showed that the reference-based assembly had significant similarities with more stickleback protein-coding genes (15,081) than the de novo assembly (13,820). Thus, we chose the reference-based assembly for downstream analyses. To further evaluate the completeness of our transcriptome, we ran CEGMA (Core Eukaryotic Genes Mapping Approach) to search for a set of 248 core proteins that are known to be present in a wide range of species (Parra et al. 2007). The results were then compared with those obtained by an independent CEGMA run using as reference the most comprehensive transcriptomic resource publicly available for the genus *Xiphophorus,* the *X. maculatus* cDNA data set (Ensembl v77) (Cunningham et al. 2014).

### Differential expression

For obtaining genewise mapping results, we kept the longest transcripts per locus against which we mapped the reads again for each sample with Bowtie2. Read counts were obtained through the software SAMtools (Li et al. 2009). In total, 188,298,275 reads were uniquely mapped (ranging from 5,069,023 to 10,764,803 per sample). The differential expression analyses were conducted in DESeq 1.10.1 (Anders and Huber 2010). Differential expression was tested among all different tissue types with or without testosterone treatment with FDR adjusted (Benjamini and Hochberg 1995) p-value threshold of 0.05. Blast2GO (Conesa et al. 2005) was used to functionally annotate the genes. The differentially expressed (DE) loci were annotated after a BLASTX search against the NCBI *nr* database (e-value < e^−6^, annotation cut-off > 55, GO weight > 5) and assignment of the corresponding gene ontology (GO) terms. Enrichment analyses, using the Fisher's exact test implemented in Blast2GO, were applied to identify significantly overrepresented GO terms comparing DEG sets to the whole assembly as a reference. Pathway analysis was performed using DAVID (Huang da et al. 2009).

## Results

### Exogenous testosterone-driven development of the sword and gonopodium

To explore gene expression profiling of the developing sword and gonopodium at the same ontogenetic stages, we applied a testosterone treatment on immature juvenile *X. hellerii* of a single brood (see details in Materials and Methods). We observed that all hormone-treated juvenile fish developed both sword-like protrusions from their ventral caudal fins (ray V7–V10) as well as the extension of gonopodial rays from the anal fin (ray V3–V5) after 5 days of treatment. The developing swords and gonopodia were clearly visible after 5 days of treatment (Fig.1).

### High-throughput transcriptome sequencing of the developing swords and gonopodia

Illumina deep sequencing yielded from 5,655,070 to 11,861,043 reads per sample, with an average of 8,381,177 read per individual (see Table1 for summary). Reference-based assembly suggested the presence of 41,360 different loci (N50: 2,570 bp). Further, 71% of our assembled transcripts had a significant similarity (assessed with BLASTN against *X. maculatus* cDNAs; e-value threshold 10^−6^) to the previously determined *X. maculatus* transcriptome. Our assembly showed a CEGMA completeness of 93.15% partial and 85.89% complete genes, while the transcriptomic data of *X. maculatus* was composed of 97.98% partial and 88.71% complete genes.

**Table 1:** Summary statistics of Illumina sequencing. Percentage of the raw reads in each tissue is shown in parentheses

	Treated				Nontreated control	
	Sword (TV)	Caudal middle (TM)	Caudal dorsal (TD)	Gonopodium (TG)	Sword (CV)	Gonopodium (CG)
Raw reads	47,457,964	46,049,838	45,121,568	42,942,650	41,862,944	48,853,452
Filtered reads	35,055,836 (73.87%)	33,711,045 (73.21%)	33,413,285 (74.05%)	31,841,652 (74.15%)	30,884,763 (73.78%)	36,241,671 (74.18%)
Uniquely mapped reads	32,917,816 (69.36%)	31,540,697 (68.49%)	31,244,476 (69.25%)	29,898,472 (69.62%)	28,875,936 (68.98%)	33,820,878 (69.23%)
Unmapped reads	2,138,020 (4.51%)	2,170,348 (4.71%)	2,168,809 (4.81%)	1,943,180 (4.53%)	2,008,827 (4.80%)	2,420,793 (4.96%)

### Gene expression profiling

**Figure 2:**
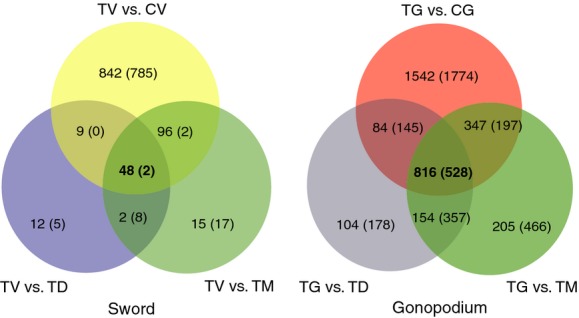
Venn diagram showing codifferentially expressed genes among different pairwise comparisons for sword and gonopodium. Numbers indicate the upregulated genes and those in parentheses the downregulated genes, respectively. TV stands for treated ventral ray (sword), TD for treated dorsal ray, TM for treated middle ray, TG for treated anal fin ray (gonopodium), CV for nontreated ventral ray in control fish, and CG for nontreated anal fin ray in control fish. *T stands for treated, C for nontreated (control), V for ventral caudal rays (sword), M for middle caudal rays, D for dorsal caudal rays, G for gonopodium (anal fin rays).

Our main goal was to characterize the gene expression profiles of the sword and gonopodial tissues during treatment-induced development. For this, we compared the gene expression patterns between different tissues within the same groups (treated or nontreated) or in the same tissues between the groups in a pairwise manner (Fig.2). The total number of differentially expressed genes (DEGs) was determined from all seven comparisons (Table2). The largest number of DEGs was observed in the gonopodium comparison between “TG” and “CG” (5433), while no DEGs were found between “TM” and “TD” groups. In the following sections, we summarized the analyzed expression patterns of the two developing male-specific organs, the sword, and gonopodium separately.

**Table 2:** DEGs found between tissues and treated or nontreated individuals

	TV	TG	TD
TM	190	3070	0
TV	–	1485	86
TG	–	–	2366
CG	–	5433	–
CV	1784	–	–

T stands for treated, C for nontreated (control), V for ventral caudal ray (sword), M for middle caudal ray, D for dorsal caudal ray.

### DEGs in the developing sword

To characterize expression profiles of the sword, we compared the gene expression of the ventral ray of the caudal fin (developing sword) with two other tissues of the caudal fin, the middle and the dorsal rays, which retain their typical caudal fin ray morphology during sexual maturation of the fish and its sword development. First, we measured the differences in gene expression patterns for the pairs of ventral rays of the caudal fin between testosterone-treated and untreated fish (TV and CV). A total of 1784 genes were differentially expressed; 995 genes were upregulated, while 789 genes were downregulated in the sword of treated fish (Table S1). In 1784 DEGs in the sword (TV and CV), we found that 372 GO terms are overrepresented compared to the reference-assembled transcriptome (Table S2).

**Figure 3:**
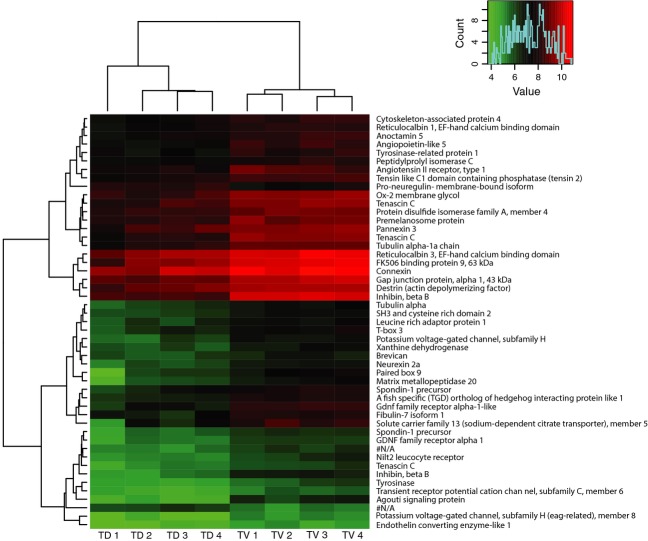
Sword-specific gene expression. Heatmap of the limited gene set for sword displaying significantly differential expression between treated dorsal (TD) and treated ventral (TV, sword) rays. Color coding represents normalized expression data (variance stabilization transformed data implemented in DESeq). *T stands for treated, V for ventral caudal ray (sword), D for dorsal caudal ray. One locus with extreme values was excluded (COL10A1: CUFF. 35437.1).

To identify which genes were differentially expressed specifically in the sword (TV), we further analyzed the gene expression in the other two tissues of the caudal fin, the middle (TM), and the dorsal rays (TD). The comparison between ventral and middle rays in treated fish (TV and TM) revealed 190 DEGs, whereas that between ventral and dorsal rays (TV and TD) found only 86 DEGs (Fig.2). Three independent pairwise comparisons resulted in 50 common DEGs (48 upregulated and two downregulated genes). This limited gene set can be considered as the sword-specific expression profile (Table3; Fig.2, Venn diagram; Fig.3, Heat map). To investigate functional classifications of sword-specific DEGs, we performed a functional enrichment analysis on the limited gene set compared to the assembled transcriptome (Fig.3). Fifteen functional categories were significantly overrepresented (FDR < 0.05) with gonadotropin secretion and follicle-stimulating hormone secretion being the most significantly overrepresented (Table S3).

**Table 3:** Sword-specific DEGs from the limited gene set (TV and CV, TV and TD, TV and TM) between tissues and treatments

ID	Gene name	Ensembl Gene id	Ensembl Gene description
Up-regulated in developing sword			
CUFF.36200.1	AGTR1	ENSXMAG00000020155	Angiotensin II receptor, type 1 [Source:HGNC Symbol;Acc:336]
CUFF.22492.1	ANGPTL5	ENSXMAG00000010840	Angiopoietin-like 5 [Source:HGNC Symbol;Acc:19705]
CUFF.15255.1	ANO5 (1 of 2)	ENSXMAG00000007021	Anoctamin 5 [Source:HGNC Symbol;Acc:27337]
CUFF.1503.1	ASIP	ENSXMAG00000012156	Agouti signaling protein [Source:HGNC Symbol;Acc:745]
CUFF.38557.1	BCAN (2 of 2)	ENSXMAG00000015085	Brevican [Source:HGNC Symbol;Acc:23059]
CUFF.11483.1	CKAP4	ENSXMAG00000001319	Cytoskeleton-associated protein 4 [Source:HGNC Symbol;Acc:16991]
CUFF.35437.1	COL10A1 (2 of 2)	ENSXMAG00000020162	Collagen, type X, alpha 1 [Source:HGNC Symbol;Acc:2185]
CUFF.40385.1	DSTN	ENSXMAG00000006139	Destrin (actin depolymerizing factor) [Source:HGNC Symbol;Acc:15750]
CUFF.24718.1	ECEL1 (1 of 2)	ENSXMAG00000002319	Endothelin converting enzyme-like 1 [Source:HGNC Symbol;Acc:3147]
CUFF.37038.1	FKBP9	ENSXMAG00000003160	FK506 binding protein 9, 63 kDa [Source:HGNC Symbol;Acc:3725]
CUFF.7252.1	GFRA1 (1 of 2)	ENSXMAG00000014650	GDNF family receptor alpha 1 [Source:HGNC Symbol;Acc:4243]
CUFF.22162.1	GJA1	ENSXMAG00000005269	Gap junction protein, alpha 1, 43 kDa [Source:HGNC Symbol;Acc:4274]
CUFF.12280.1	INHBB (1 of 2)	ENSXMAG00000006435	Inhibin, beta B [Source:HGNC Symbol;Acc:6067]
CUFF.12283.1	INHBB (1 of 2)	ENSXMAG00000006435	Inhibin, beta B [Source:HGNC Symbol;Acc:6067]
CUFF.27942.1	KCNH8	ENSXMAG00000000864	Potassium voltage-gated channel, subfamily H (eag-related), member 8 [Source:HGNC Symbol;Acc:18864]
CUFF.27947.1	KCNH8	ENSXMAG00000000864	Potassium voltage-gated channel, subfamily H (eag-related), member 8 [Source:HGNC Symbol;Acc:18864]
CUFF.25856.1	LURAP1 (1 of 2)	ENSXMAG00000002255	Leucine rich adaptor protein 1 [Source:HGNC Symbol;Acc:32327]
CUFF.17142.1	MMP20	ENSXMAG00000001558	Matrixmetallopeptidase 20 [Source:HGNC Symbol;Acc:7167]
CUFF.26478.1	PANX3	ENSXMAG00000006184	Pannexin 3 [Source:HGNC Symbol;Acc:20573]
CUFF.33889.1	PAX9	ENSXMAG00000015762	Paired box 9 [Source:HGNC Symbol;Acc:8623]
CUFF.15331.1	PDIA4	ENSXMAG00000018874	Protein disulfide isomerase family A, member 4 [Source:HGNC Symbol;Acc:30167]
CUFF.25172.1	PMEL (1 of 2)	ENSXMAG00000003314	Premelanosome protein [Source:HGNC Symbol;Acc:10880]
CUFF.15589.1	PPIC	ENSXMAG00000015311	Peptidylprolylisomerase C (cyclophilin C) [Source:HGNC Symbol;Acc:9256]
CUFF.25279.1	RCN1	ENSXMAG00000014239	Reticulocalbin 1, EF-hand calcium binding domain [Source:HGNC Symbol;Acc:9934]
CUFF.11860.1	RCN3	ENSXMAG00000002579	Reticulocalbin 3, EF-hand calcium binding domain [Source:HGNC Symbol;Acc:21145]
CUFF.25595.1	SLC13A5 (1 of 2)	ENSXMAG00000004065	Solute carrier family 13 (sodium-dependent citrate transporter), member 5 [Source:HGNC Symbol;Acc:23089]
CUFF.5285.1	STAC2 (2 of 2)	ENSXMAG00000002187	SH3 and cysteine rich domain 2 [Source:HGNC Symbol;Acc:23990]
CUFF.26167.1	TBX3	ENSXMAG00000005372	T-box 3 [Source:HGNC Symbol;Acc:11602]
CUFF.36189.1	TENC1 (2 of 2)	ENSXMAG00000018693	Tensin like C1 domain containing phosphatase (tensin 2) [Source:HGNC Symbol;Acc:19737]
CUFF.29119.1	TNC (1 of 2)	ENSXMAG00000004876	Tenascin C [Source:HGNC Symbol;Acc:5318]
CUFF.28413.1	TNC (2 of 2)	ENSXMAG00000018617	Tenascin C [Source:HGNC Symbol;Acc:5318]
CUFF.28417.1	TNC (2 of 2)	ENSXMAG00000018617	Tenascin C [Source:HGNC Symbol;Acc:5318]
CUFF.23730.1	TRPC6 (1 of 2)	ENSXMAG00000010842	Transient receptor potential cation channel, subfamily C, member 6 [Source:HGNC Symbol;Acc:12338]
CUFF.26841.1	TYR (1 of 2)	ENSXMAG00000011209	Tyrosinase [Source:HGNC Symbol;Acc:12442]
CUFF.25560.1	TYRP1 (2 of 2)	ENSXMAG00000004910	Tyrosinase-related protein 1 [Source:HGNC Symbol;Acc:12450]
CUFF.4663.1	XDH	ENSXMAG00000003461	Xanthine dehydrogenase [Source:HGNC Symbol;Acc:12805]
CUFF.11637.1	#N/A	ENSXMAG00000005039	Fibulin-7 isoform 1
CUFF.12274.1	#N/A	#N/A	#N/A
CUFF.17007.1	#N/A	ENSXMAG00000018184	A fish specific (TGD) ortholog of hedgehog interacting protein like 1
CUFF.22535.1	#N/A	ENSXMAG00000011006	Neurexin 2a
CUFF.23770.1	#N/A	#N/A	NILT2 leucocyte receptor
CUFF.31467.1	#N/A	ENSXMAG00000015953	Tubulin alpha
CUFF.31478.1	#N/A	ENSXMAG00000015953	Tubulin alpha-1a chain
CUFF.33581.1	#N/A	ENSXMAG00000003979	OX-2 membrane glycol
CUFF.4179.1	#N/A	#N/A	GDNF family receptor alpha-1-like
CUFF.7419.1	#N/A	ENSXMAG00000000509	Connexin
CUFF.8191.1	#N/A	ENSXMAG00000006973	Spondin-1 precursor
CUFF.8192.1	#N/A	ENSXMAG00000006973	Spondin-1 precursor
Down-regulated in developing sword			
CUFF.35587.1	#N/A	#N/A	Pro-neuregulin- membrane-bound isoform
CUFF.12776.1	#N/A	#N/A	#N/A

Pathway analysis and functional annotation results based on the comparison between the sword (TV) and dorsal caudal (TD) fin in the treated fish showed that two of the upregulated coloration related pathways are overrepresented in the developing sword. One is tyrosine metabolism (has00350) and the other is melanogenesis (has04916) (see Figure S1). Moreover, the functional annotation results revealed that many of the coloration-related GO terms such as melanosome, melanogenesis, pigmentation, tyrosinase, and melanin biosynthesis are overrepresented (Figure S2).

### DEGs in the developing gonopodium

Using the same comparison scheme as for the sword, we determined DEGs in the development of the gonopodium. Expressed genes in gonopodium tissues (TG and CG, see Fig.1) between treatment and control fish were compared. A total of 5433 genes were found to be differentially expressed: 2789 genes of those were upregulated and 2644 were downregulated in TG compared to the nonmaturing control fish (CG) (Table S4). The GO term enrichment analysis of those 5433 genes revealed that 240 functional categories are significantly overrepresented compared to the assembled transcriptome (Table S5). Similar to our analyses on the sword (TV), we conducted further analyses restricted to the gonopodium-specific DEGs. We found 816 upregulated and 528 downregulated genes in TG compared to CG, TM, and TD (Figs.2, Table S6). In the gonopodium-specific DEGs, we found 35 overrepresented GO terms compared to reference transcriptome (Table S7).

### Gene expression patterns shared between the sword and gonopodium

To identify the common molecular mechanisms that are found during both sword and gonopodium development, we compared the lists of DEGs and enriched GO terms from the comparisons of “TV and CV” and “TG and CG.” We found that 643 upregulated and 610 downregulated genes are shared (Table S8). Independent GO terms enrichment analyses showed that 132 of enriched terms are common, while overrepresented GO terms for only in sword and gonopodium are 240 and 108, respectively (Table S9).

### Gene expression differences in the developing anal and ventral caudal fins

To identify which genes were initially differentially expressed in untreated (control) ventral caudal (CV) and gonopodium (CG) (control anal fins) before the sword and gonopodium visibly developed, we compared the expression patterns between those two tissues solely in control fish. 115 of the DEGs were upregulated in the control ventral caudal fin (CV), while 39 of the DEGs were upregulated in the control gonopodium (anal fin) (CG) (Table S10). We further compared those DEGs (CV and CG) to ones found in the developing sword (TV and CV) to test whether the initial difference of the expressed genes due to tissue (ventral caudal and anal fin) specificity is increased, decreased, or completely lost during sword and gonopodium development in the treated fish. We found that 27 genes of 115 upregulated DEGs in CV were further upregulated during the development of the sword (TV and CV). Those interesting genes include eight collagen genes (*col1a1*, *col1a2*, *col5a2*, *col5a3*, *col10a1*, *col12a1*, *col14a1*, *col27a1*), one *Hox* gene (*hoxc13*) and *insulin-like growth factor 2* (*igf2*) (Table S10). In the control (not treated with testosterone) anal fin (CG), 12 of 39 upregulated DEGs retained their gene expression differences in the developing gonopodia as well (TG and CG), including four *Hox* genes (*hoxa9*, *10*, *11*, and *lhx9*) (Table S10). These findings suggest that pre-existing differential expression patterns between anal and ventral caudal fins (tissue-specific variation) during early ontogeny are partly further maintained when they became transformed into the male-specific organs.

## Discussion

This study provides the first comprehensive catalog of the genes activated in two developing male-specific traits, the gonopodium and the sword. Our analyses identified hundreds of genes linked to the development of these two evolutionary novelties. That so many genes show expression differences surprised us initially, but might reflect the complexity of the biological processes involved in the transformation of a simple fin into a sperm-transfer organ or a colorful exaggerated male ornament. Genes activated (positively or negatively) in the developing tissues can be (1) hormone responsive genes involved in the initiation and continuation of the development of traits during sexual maturation; (2) genes regulating cellular processes during postembryonic development; and (3) genes contributing to downstream morphological changes such as cell proliferation, outgrowth of fin rays, addition of new segments, and coloration. We find many genes already known to be involved in those significant biological functions and numerous male-biased or sex-specific genes as well.

Effects of sex on testosterone response would be trivial in this experiment. It is not feasible to determine sex of juveniles before sexual maturation as gonad development and sexual differentiation have not yet occurred at the age of 3 months. The amount of testosterone in this experiment would be sufficient to eliminate the original sex differences. If putative male or female juveniles responded differentially to the hormone, one might expect also that the hormone-induced sword and gonopodium should differ between the sexes. However, all hormone-induced swords and gonopodia developed very similarly in terms of their morphology and timing of development. Furthermore, we observed that all induced swords and gonopodia are morphologically almost identical among juveniles during the extended duration of testosterone treatment at 18 days (induced sword and gonopodium development is completed at 18 days of treatment) (Offen et al. 2008, 2009). However, the possibility of functional sex reversal by exogenous steroid hormone cannot be completely ruled out as this phenomenon is not rare in teleost fishes (Pandian and Sheela 1995). If sex hormones exclusively determined the sex and sexual differentiation in this fish species, hormone-treated fish in our experiments would be expected to be all males.

### Genes for hormone response: the androgen signaling pathways

The differential expression of hormone responsive genes such as androgen and/or estrogen would be expected in testosterone-induced swords and gonopodia in juveniles. Sex hormones play an important role in the development of secondary sexual traits. Artificially induced gonopodia by exogenous testosterone in juveniles, even in females of other poeciliid fish such as *Gambusia affinis*, suggests that androgen signaling is involved in the development of the gonopodium. Therefore, androgen receptors would be the first genes to be considered as key genes to orchestrate the complete network leading to the differential expression of all other genes in the development of both traits. However, differential expression of androgen receptors was not found between the developing sword (TV) and untreated control fin (CV). But, differentially expressed androgen induced 1 (AIG-1) and many other downstream targets of androgen signaling pathways such as *fgf7* and *fgf16* in the developing sword (TV) were found (Table S1) and might indicate an activation of androgen signaling. Similarly, differential expression of the androgen receptor gene was not observed between the developing gonopodium (TG) and untreated control anal fin (CG) (Table S4). This could be a sign of no expression differences of androgen receptors in the developing gonopodium or, alternatively, indicate on equal expression of androgen receptor in both tissues. The latter is more likely as our previous study using in situ hybridization (Offen et al. 2013) detected the expression of androgen receptor *β* (*arβ*) in the untreated control anal fin. This hypothesis is further supported by our transcriptome data demonstrating that differential expression of androgen receptors between the anal fin (CG) and the ventral caudal fin (CV) in untreated control fish (Table S10). It suggests that the regulation of androgen receptor originally differs between those two tissues before the development of the sword and the gonopodium. In situ hybridization showed that upregulation of androgen receptor *β* (*arβ*) was detected constantly in both developing gonopodia and untreated control fins during development, whereas swords showed a more dynamic and complicated expression patterns of *arβ*, for example, its expression depends upon timing and the position of rays. Taken all information into consideration, it seems likely that androgen signaling is differentially regulated in the developing sword and gonopodium. The dynamic nature of *arβ* expression in the sword could explain why sword develops from the ventral caudal fin only.

### Male-biased, sex differentiation, sex determination genes

On a related note, the differential expression of male-biased, sex differentiation or sex determination genes is expected as these traits are male-specific and developed during sexual maturation in natural condition. We have indeed found several genes that were previously identified as having sex-specific gene expression patterns. For example, *cyps* and *gpx 7, 8* (Tables S4 and S8) were differentially expressed in the developing sword and/or gonopodium. *Cyps* plays a role in catalyzing the oxidation of organic substances, and *gpx 7*, *8* are antioxidant genes (Zheng et al. 2013). The sword-specific gene list (Table3) shows several sex-specific development-related genes such as *col10a1* and *inhbb*. *Inhbb* has been suggested to regulate the sexually dimorphic differentiation of gonads. It contributes to the formation of the coelomic vessel, which is critical for testis development, while it is suppressed by *Wnt4* in the ovary (Yao et al. 2006). *Wnt4* – a well-known gene for its role in female sex development (Chassot et al. 2012; Li et al. 2014) – was downregulated in both the sword and the gonopodium, and it has previously been suggested to be a female sex determination gene (Forconi et al. 2013). It appears that female-biased genes are downregulated or inhibited. Another interesting gene is *Sox9*, which has been identified as a sex determination gene (Sekido and Lovell-Badge 2008). Its conserved role during gonad development in vertebrates is known (Yokoi et al. 2002), and it is also associated with testis differentiation in mouse (Wainwright et al. 2013) and other mammals (Barrionuevo et al. 2012). In our study, several *sox* genes are differentially expressed in the sword (*sox5*, and *10* are upregulated, while *sox2*, *3*, *9* are downregulated) (Table S1) and in the gonopodium (*sox4*, *6*, *18* upregulated, while *sox3*, *9*, *13* downregulated) (Table S4).

However, regarding the potential role of these genes as master regulators for sword and gonopodium development or sexual differentiation, it should be noted that the developmental processes of hormone-induced sword/gonopodium might still differ from those of naturally developed ones. So far, too little is known about sex determination and differentiation in *Xiphophorus*. In general, sexual differentiation could be regulated by sex chromosomes in a cell-autonomous fashion or by a sex-specific hormone signal received from the gonads or other tissues (Bear and Monteiro 2013). It is likely that sex-biased and/or sex determination genes are involved in the development of hormone-induced sword/gonopodium. Sex-biased or sex-specific genes that were identified in this study should be the focus of more future attention in an effort to investigate whether they are involved in the origin of sexual dimorphic traits in *X. hellerii*.

### Shared genes between the development of the sword and the gonopodium: signals of co-option

Commonly expressed genes during sword and gonopodium development offer intriguing insight into the potentially shared genetic mechanisms underlying both of these two types of male-specific traits. Previous studies already suggested that genes expressed in the evolutionarily “older” trait, gonopodium, might be co-opted during the ontogeny and evolution of the sword, a more recent evolutionary innovation in the genus *Xiphophorus* (Zauner et al. 2003; Offen et al. 2008). Indeed, our transcriptome analysis supports this idea as good portions of genes (about 70% of DEGs) are shared between the sword (TV and CV) and gonopodium (TG and CG) (see Tables S8 and S9 for the full list). Further investigation is required for a deeper understanding of these potentially common genetic regulatory pathways and the role that they play in each of the gonopodium and sword developmental processes.

Our data also suggest that the development of both traits in swordtails seems to be a result of pleiotropic effects of several genes and/or some that are co-opted from many embryonic developmental genes. Shared genes by both male-specific traits are involved in many developmental processes (e.g., tissue development, skeletal system development, collagen fibril organization, system development, and various organ development, etc.) and morphogenesis (e.g., organ, anatomical and skeletal system) related biological functions (see full list, Table S9). This list includes many of those pathways that are involved in embryonic developmental gene networks (i.e., limb development, organ development). The recruitment of already existing gene networks using “gene network co-option” has been suggested to be a usual way for the development and evolution of novel traits (Fraser et al. 2009; Monteiro and Podlaha 2009). Recently, the co-option of a gene network has been suggested to underlie the origin of a novel trait – for example, red patches of pigmentation on butterfly wings may have resulted from the co-option of eye-developmental genes (i.e., *optix*) (Monteiro 2012). Such cases are also observed in the development of morphologically specific organs or sexually selected traits in other animal groups (Moczek and Rose 2009). For example, *Hox* genes are well-known principal transcriptional regulators of animal body regionalization in embryonic development (Meyer and Málaga-Trillo 1999; Kopp 2011; Tanaka et al. 2011). *Hox* genes have also been suggested to be key-players in the development and evolution of novel complex traits such as beetle horns (Wasik et al. 2010), male genitalia (i.e., imaginal discs) (Estrada et al. 2003), and a secondary sexual trait – sex combs in *Drosophila* species (Barmina and Kopp 2007). We also found many *hox* genes (i.e., *dlx*, *lhx9*, *satb2*, *zhx2* and etc.) to be commonly expressed in both evolutionary novelties under consideration here, the sword and the gonopodium. Taken together, it is likely that genetic regulatory networks were co-opted during gonopodium development and evolution and were subsequently deployed as well in the later evolution of another novelty of the sword.

### Genes contributing to downstream morphological changes

#### Gene expression in the sword

A large number of genes were found that appear to be involved in the development of the sword. In the comparison between TV and CV, we found many genes and related functions that might be expected from the complicated morphological and biological changes occurring during its development. Genes or pathways involved in early embryonic development are activated again during the metamorphosis of fin rays into the sword. The analyses revealed various embryonic organ development-related functions such as chordate embryonic development, embryonic morphogenesis, embryonic organ development, embryonic skeletal system development, and in utero embryonic development. Among the sword-specific genes (Table3), several genes with crucial roles during embryonic development and organogenesis such as *pax9* and *tbx3* are found (Table3). For instance, *Pax9* is known to play pleiotropically essential roles in the development of the craniofacial skeleton, the dentition (Peters et al. 1999), and tooth morphogenesis in mice (Kapadia et al. 2007). *Tbx3* is also thought to play a role in the posterior/anterior axis of tetrapod forelimb (Gibson-Brown et al. 1996), heart development (Ribeiro et al. 2007), and genital development (Ballim et al. 2012). However, a function for them in the development of the sword had not been suggested before. This indicates that even though the sword development happens at a postembryonic stage, it reemploys the same genetic toolkit used for many fundamental processes during early embryonic development.

Many genes (described by their respective GO terms) that are expected to be responsible for morphological changes of the developing sword are indeed activated (Table S2). The development of the sword includes various morphological changes such as outgrowth of fin rays and addition of segmentation. It should also be noted that all these genes and functions are simultaneously activated at early stages of the sword's development even before all characteristics of the sword such as the elongation of fin rays, coloration, and segmentation have appeared.

#### Why does only the ventral caudal fin develop into sword?

Dorsal and ventral parts of the caudal fin are morphologically very similar (e.g., number of rays and segments) before the sword develops. Yet, only the ventral caudal fin rays undergo a transformation into a sword during sexual maturation. Interestingly, through testosterone treatment also the dorsal caudal fin rays are occasionally induced to produce a sword-like protrusion, although it does not develop into a fully developed sword even with prolonged exposure to testosterone (Eibner et al. 2008). Therefore, DEGs in the comparison between the ventral and dorsal caudal fins (TV and TD) might inform which genes are functionally necessary for the origination of the fully developed sword. We found 71 upregulated and 15 downregulated genes in the ventral caudal fin (sword) compared to dorsal portion of the caudal fin (Table S11). Most of the upregulated genes are the same as those in the restricted gene list (Table3). However, several genes such as *col10a1, fndc7*, *clec19a*, *anionic trypsin-2-like* (collagen catabolic process)*, zona pellucida-like domain-containing protein 1-like* are also overexpressed in the ventral caudal fin compared to the dorsal portion of the caudal fin (Table S11). Those genes are generally known to be involved in cell proliferation and growth. This could be simply explained by the fact that the ventral caudal fin is growing faster compared to the dorsal fin. However, it seems worthwhile noting that compared to other tissue comparisons (e.g., TD vs. CV, TV vs. CV), a rather smaller number of genes are differently expressed between the dorsal (TD) and the ventral caudal fin (TV) in the testosterone-treated fish (Table2), probably indicating their similarities in their “ontogenetic potential”. Gene expression of those genes might contribute to the transformation of the ventral rays into the sword, although it is not clear how far “upstream” these genes are in the “command-chain” permitting the sword transformation or even inducing it. Testosterone treatment induces only a small protrusion in the dorsal rays of the caudal fin in *X. hellerii* (Sangster 1948; Dzwillo 1964; Offen et al. 2008), and we found earlier that the transplantation of a single ventral sword ray to the dorsal caudal fin induced an ectopic sword dorsally (Eibner et al. 2008). Apparently the sword signal is carried with the transplanted ventral ray to new dorsal caudal fin location. Therefore, our results suggest that rather small changes of gene expression in the ventral caudal fin rays lead to the different fate of dorsal and ventral caudal fin rays during sexual maturation and the transformation of simple fin rays, into structures that as a composite make up the sword.

### Color genes in the sword (The colorful sword)

The sword is a composite trait consisting of a yellow coloration and a black ventral margin with remarkable inter- and intraspecific variation of coloration (Basolo 1991; Meyer 1997). Gene lists from other comparisons provide further detailed information of color gene expression in the developing sword. Several upregulated melanogenesis-related genes (the regulation of melanocyte-related pigmentation) such as *asip*, *pmel*, *tyr*, *mc1r,* and *mlph* in the developing sword (Table S1) would be responsible for black coloration. Genes involved in expressing yellow or orange coloration are also upregulated in the developing sword. For example, *xdh* is known to regulate the synthesis of pigments for yellow or orange coloration found in xanthophores (Oliphant and Hudon 1993).

Interestingly, in our study, the color genes show slightly different expression patterns based on the different comparisons. For example, the expression of *xdh* and *asip* seems to be sword-specific as they are upregulated in the developing sword (TV) compared to the treated caudal fins (TM and TD) and untreated caudal fins (CV) (Table3). No differential expression between the middle caudal fin (TM) in treated fish and the ventral caudal fin (CV) in control fish further support the sword-specific gene expression of *xdh* and *asip*. On the other hand, *mc1r* shows differential expression only in a comparison between the sword (TV) and nontreated ventral caudal fin (CV) (Table S1).

Other yellow coloration carotenoid-related color genes [i.e., *bco2*] seem to be more influenced by a hormonal response as they are upregulated in the treated middle caudal fin (TM) compared to untreated ventral caudal fin (CV). This finding indicates that some color genes (i.e., *bco2*) are testosterone hormone-induced genes, while others (e.g., *xdh* and *asip*) are more tissue- or sword-specific genes. In short, our data show that several color genes are expressed during early sword development. These results suggest that genes down- or upstream of color genes should be carefully considered to understand the whole process of the transformation of the ventral rays of the caudal fin into a sword.

#### Gonopodium gene expression

As in the sword, active genes with functions such as responses to hormone stimuli, embryonic organ development, and organ morphogenesis are also expressed in the developing gonopodium. Interestingly, our results show that the number of DEGs in the developing gonopodium is approximately three times greater than that in the developing sword. Obviously, the gonopodium is a more complex structure than the sword and hence, this result would make sense as the size of genetic networks can be translated into more diverse morphological structures as, for example, much fewer genes are involved in hair coloration than more complex traits such as craniofacial shape in humans (Claes et al. 2014). However, the earlier onset of the gonopodium development relative to the sword development might simply add to the observed result that more genes are being activated in the developing gonopodium than in the sword.

In addition to a larger number of genes expressed in gonopodium, there are several unique characteristics in its gene expressions, compared to the sword (Table S9). For example, GO terms for dorsal/ventral pattern formation and regionalization were found only in the gonopodium, but not in the sword (Table S9). The gonopodium develops a very specific proximal–distal (PD) axis difference due to its specialized distal structures such as hooks and a claw (Figure S3). Although in the early gonopodium those traits are not developed yet, the specific pattern gene expression might determine cell fate in the early developmental stages. Activation of the *Wnt*-signaling pathway is a well-known biological pathway that regulates PD axis formation. We found four *wnt* genes (*wnt4*, *7a*, *9b,* and *11*) to be downregulated in the developing gonopodium (TG) compared to anal fin in untreated fish (CG) and particularly the *wnt7* downregulation compared to other tissues in the treated fish might be worth following up on as wnt-genes are often implicated to play a role in the development of complex features and adaptive structural differences such as the shape of the neurocranium in fish (Parson et al. 2014) and tetrapods (Claes et al. 2014).

If those genes were activated in anal fin in untreated fish (CG), we would expect to detect a downregulated expression in the developing gonopodium (TG) in treated fish. Alternatively, the expression of those genes could be suppressed in male traits induced by testosterone treatment as, for example, *wnt4* genes are known to be one of the female sex determination genes in fish (Forconi et al. 2013). Homologs of *fgf9* and *sry* – known to regulate mouse testis anterior and posterior determination (Hiramatsu et al. 2010) – are also upregulated in the developing gonopodium (Table S4). In future studies, investigations of gene expression patterns in the developing gonopodium and sword throughout different ontogenetic stages would allow for differentiating gonopodium-specific genetic networks from sword. And, once technically feasible, knock-down or knock-out experiments might help to establish functional roles more clearly (Kratochwil and Meyer 2015a,b).

### Are common sets of genes differentially expressed in male exaggerated ornaments?

Like swordtails, many other animal taxa have developed male exaggerated ornaments (or weapons) that are known to play a role in intra- and intersexual selection. Considering the sword gene list, we were interested in whether there are shared or conserved genetic pathways between the sword and other sexually selected male ornaments. Interestingly, several genes in the sword gene list are expressed or involved during development of other male exaggerate ornaments (weapon) or sexually selected traits.

For instance, *fork head* (*fkh*) genes (e.g., *foxs*) are involved in sex combs as natural targets of *scr* in fruit flies (Ryoo and Mann 1999), *crol* and *cdc2* in eye span in stalk-eyed flies (Baker et al. 2009), *dll* or *dlx* in sex comb in *Drosophila* and butterfly eyespot size (Dworkin 2005; Monteiro et al. 2013), *bmp2* in comb mass in chickens (Johnsson et al. 2012), and *IGF* pathways and *InRs* in the rhinoceros beetle horns (Emlen et al. 2012; Lavine et al. 2013). These genes or its homologs such as several *fork head box* genes (i.e., *foxd3*), zinc finger proteins as the homologs of *crol, cdcs* (e.g., *cdc7*, *16*, *20*, *27,* and *34*), *dlxs* (*dlx1*, *2,* and *4*), *bmps* (*bmp1*)*,* and *IGF* pathway-related genes (*igf2*, *igfbp1, 2*, *4*, *6,* and *irs2*) were found to be expressed in the developing sword and gonopodium (Tables S1 and S4). Nevertheless, we cannot rule out the possibility that the common set of these genes simply function as a morphogens and/or growth factors for the morphological changes ensuring in the developing sword and gonopodium. However, for instance, it was shown that the *IGF* pathway might be responsible for exaggeration of the structure and byproduct of the growth mechanism in beetle horn (Emlen et al. 2012). The fact that our identified candidate genes belong to the same gene families and also play a role in male exaggerated ornaments might suggest their potential role in different sexually selected traits across different animal lineages. This hypothesis clearly needs further study, but is an exciting finding that deserves follow-up work.

## Conclusions

Comparative RNA sequencing of evolutionary novelties such as the gonopodium and the sword provided insights into the set of genes that are involved in the development of these male-specific traits. A large number of expression-biased genes were identified in this study: 5433 and 1784 of DEGs for the gonopodium and the sword, respectively highlighting the massive changes that are taking place during the transformation of the anal fin into a gonopodium during the sexual maturation. The genes identified here and the mode of regulatory evolution suggested by our results represent the basis for further research on understanding the molecular mechanisms underlying the development of male-specific traits in swordtails, in other male-specific traits and even in sexually selected traits in animals generally.

## Data accessibility

The short read DNA sequences for this study have been deposited in the European Nucleotide Archive (ENA) under the accession code PRJEB8012.

## Supporting Information

**Figure S1.** Pathway analysis based on the comparison between the sword (TV) and dorsal caudal (TD) fin in the treated fish.

**Figure S2.** Functional annotation based on the comparison between the sword (TV) and dorsal caudal (TD) fin in the treated fish.

**Figure S3.** Structure of the gonopodium in adult *Xiphophorus hellerii* (Scale bars: 500 µm) (This figure is modified from (Offen et al. 2013)).

**Table S1.** Differentially expressed genes (DEGs) in the sword (TV) compared to the non-treated ventral caudal fin (CV). **Table S2.** Functional annotation clustering for DEGs in the sword (TV) compared to the non-treated ventral caudal fin (CV). **Table S3.** Functional classifications of sword-specific DEGs. **Table S4.** Differentially expressed genes (DEGs) in the gonopodium (TG) compared non-treated anal fin (CG). **Table S5.** Functional annotation clustering for DEGs in the gonopodium (TG) compared non-treated anal fin (CG). **Table S6.** Gonopodium-specific differentially expressed genes. **Table S7.** Functional classifications of gonopodium-specific DEGs. **Table S8.** Commonly up-regulated and down-regulated genes between the sword (TV vs. CV) and gonopodium (TG vs. CG). **Table S9.** Functional classifications of shared and sword-/gonopodium- specific DEGs. **Table S10.** Differentially expressed genes from the comparison of the non-treated ventral caudal (CV) with the non-treated anal (CG) fin rays. **Table S11.** Differentially expressed genes from the comparison of the treated ventral (TV) with the treated dorsal (TD) fin rays.
