# Supplementary figures and images for: Transcriptomics of two evolutionary novelties: how to make a sperm-transfer organ out of an anal fin and a sexually selected “sword” out of a caudal fin
Source: Ecol Evol. 2015 Jan 23;5(4):848–64. doi: 10.1002/ece3.1390 (PMC4338968; doi:10.1002/ece3.1390)

### pathways (up)

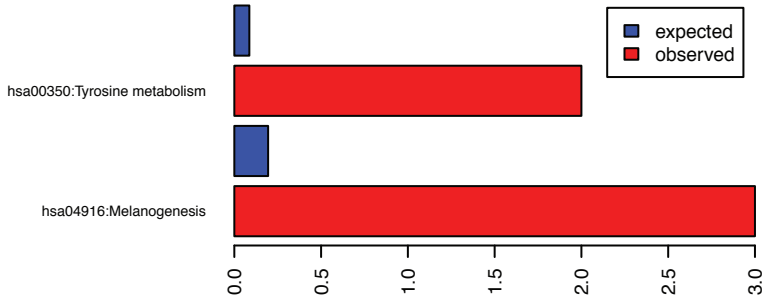

Supplement: Supplementary file 1 [file ece30005-0848-sd1.pdf]

functional annotation (up + down)

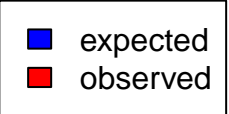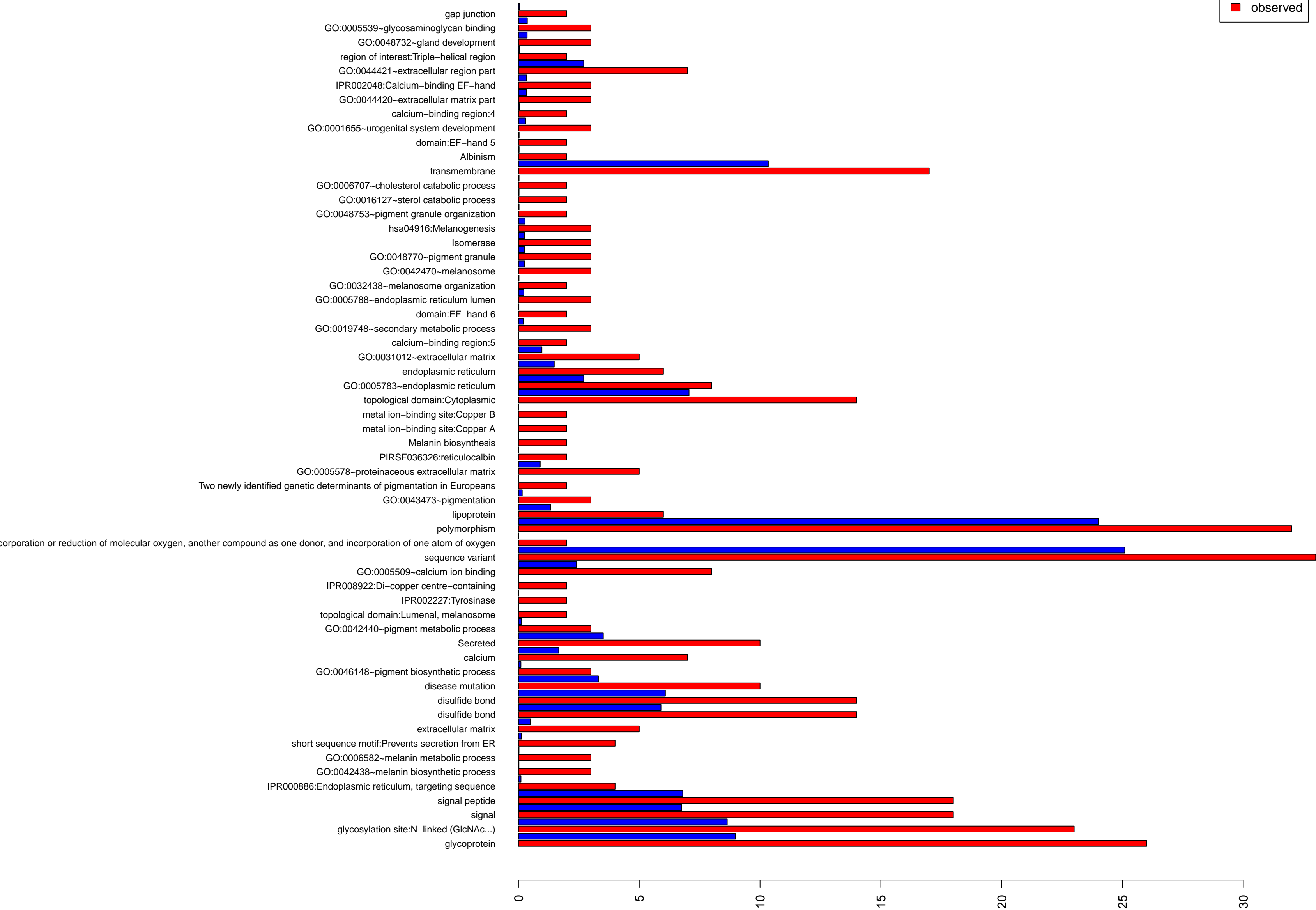

Supplement: Supplementary file 2 [file ece30005-0848-sd2.pdf]

Ray 5

Ray 4

Ray 3

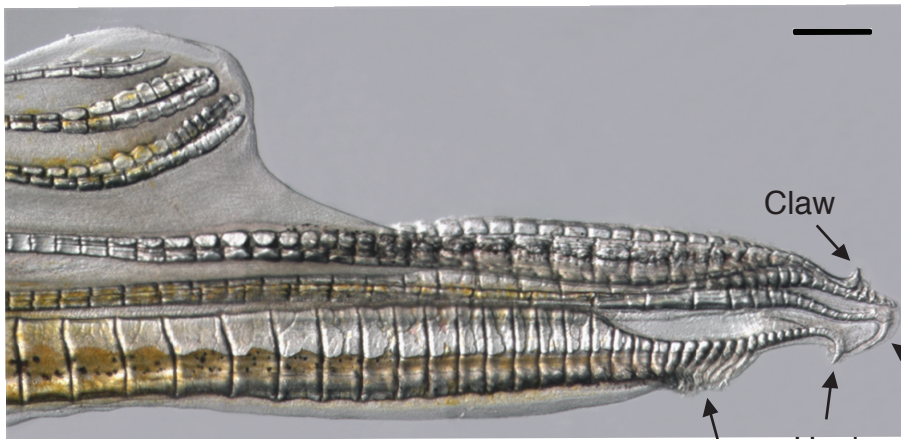

Spine

Hook

Ramus

Supplement: Supplementary file 3 [file ece30005-0848-sd3.pdf]
